# Development of a plasma- and albumin -free recombinant von Willebrand factor

**DOI:** 10.1055/s-0037-1617202

**Published:** 2009-09

**Authors:** P. L. Turecek, A. Mitterer, H. P. Matthiessen, H. Gritsch, K. Varadi, J. Siekmann, K. Schnecker, B. Plaimauer, M. Kaliwoda, M. Purtscher, W. Woehrer, W. Mundt, E.-M. Muchitsch, T. Suiter, B. M. Ewenstein, H. J. Ehrlich, H. P. Schwarz

**Affiliations:** 1 Baxter BioScience, Vienna, Austria

**Keywords:** Recombinant von Willebrand factor, von Willebrand disease, hemophilia, substitution therapy

## Abstract

Baxter has developed a recombinant therapy for treating von Willebrand‘s disease. Recombinant VWF is co-expressed with the rFVIII in CHO cells used to produce the rFVIII product Advate. This rVWF is used as a drug component for a rVWF-rFVIII complex drug product. CHO cells produce partially processed and partially un-processed rVWF still containing the pro-peptide. In order to make a consistent preparation containing mature and processed rVWF only, rVWF is exposed to recombinant furin to remove the pro-peptide. Recombinant VWF and furin are produced under serum- and protein-free conditions. It is highly purified by a series of chromatographic steps and formulated in a protein-free buffer and has a homogeneous multimer distribution. The specific activity is higher in rVWF than in commercial plasma-derived VWF-FVIII complex products. SDS agarose electrophoretic analysis shows the presence of ultra-high molecular weight multimers. The FVIII-binding capacity and affinity of rVWF to FVIII is comparable to VWF in plasma. Carbohydrate analysis shows an intact glycosylation pattern. Recombinant VWF binds to collagen and promotes platelet adhesion under shear stress. It stabilizes endogenous FVIII in VWF-deficient knock-out mice as seen by a secondary rise in murine FVIII.

Von Willebrand factor (VWF) is a multimeric adhesive glycoprotein which has a dual function in hemostasis. It

- mediates platelet adhesion at sites of vascular injury, which is necessary for primary hemostasis, and
- stabilizes factor VIII in the circulation.

The pathophysiological significance of these different biological functions is evident in von Willebrand's disease (VWD), the most common hemorrhagic disorder, affecting 1% of the population. Approximately 1% of patients with VWD have severe VWF deficiency resulting in defective platelet adhesion, secondary factor VIII (FVIII) deficiency, and a prolonged bleeding time. These patients can only be treated effectively with VWF concentrates 1 , 2 , 3 , 4 , 5 .

A variety of plasma-derived concentrates (with or without FVIII) are available for treating VWD. Development of a recombinant VWF (rVWF) manufactured and formulated in the absence of animal or human plasma proteins will increase the choice of treatment options, but also make VWD treatment independent of blood supply.

## Methods

### Plasma-derived VWF

Plasma-derived VWF (pdVWF), required as a control article for biochemical analysis, functional characterization and in vivo studies was purified in-house from the cryoprecipitate of pooled human plasma by a sequence of conventional chromatography steps and was formulated as for rVWF. Two commercially available pdVWF products, Haemate HS (ZLB Behring, Germany; lot 56966411A) and Wilfactin (LFB, Les Ulis, France; lot 33103–22238103) were used in control experiments.

### Biochemical methods

VWF : Ag ELISA was performed with a commercial polyclonal rabbit anti-human VWF antibody (Dako, Glostrup, Denmark) using the single incubation multilayer immune technique (SIMIT). VWF ristocetin cofactor (VWF : RCo) activity was measured with the BCS (Behring Coagulation System) analyzer (Siemens, Marburg, Germany) according to the manufacturer’s instructions using lyophilized von Willebrand reagent containing stabilized platelets and ristocetin A (Siemens). The FVIII-binding capacity of VWF (VWF : FVIIIB) was measured by an ELISA combined with a chromogenic assay (ECA) and by a Biacore 3000 system (Biacore, Uppsala, Sweden) using a VWF-coated CM5 chip. SDS-polyacrylamide gel electrophoresis (SDS-PAGE) was performed under reducing conditions using gradient (3–8%) Tris-acetate gels followed by Coomassie staining. VWF multimer analysis was performed using low- and high-resolution horizontal SDSagarose gel electrophoresis followed by immunostaining with a polyclonal rabbit anti-human VWF antibody 6 , 7 . Carbohydrate analysis was performed by separating PNGaseF-released N-glycans using Dionex technology. VWF-mediated platelet adhesion to collagen under shear stress was determined using a parallel-plate perfusion chamber coated with fibrillar collagen type 1.

### Studies in VWF-deficient mice

Pharmacokinetic analysis was performed in VWF-deficient mice 8 by i.v. application of 200 U VWF : Ag/kg. Citrated plasma from five animals was collected at each time point by heart puncture and analyzed for VWF : Ag (ELISA) and FVIII activity (chromogenic assay). The half-life of VWF was calculated by a single exponential fit between data points from 3 to 24 hours. The area under the curve for the rise in endogenous FVIII was calculated and included baseline mouse FVIII.

## Results

### Up- and downstream processing

**Fig. 1:**
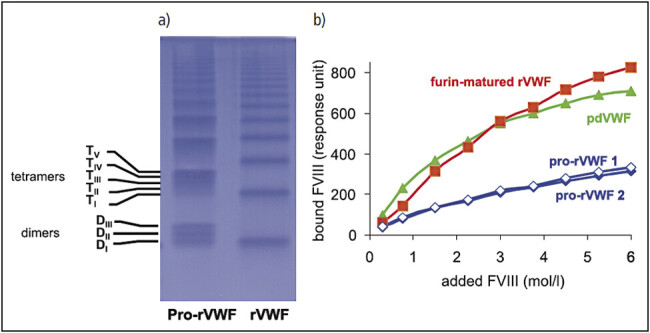
**a)** Pro-rVWF contains hetero- and homo-polymers of pro- and mature VWF (left lane), purified rVWF is fully processed (right lane) **b)** FVIII binding of rVWF is similar to that of pdVWF and several-fold higher than that of pro-rVWF

Recombinant VWF is co-expressed with recombinant FVIII in CHO cells. The material obtained from column flow-through of the FVIII capture step, containing pro-VWF, is the starting material for rVWF. It is an intermediate of the manufacturing process of the commercial rFVIII product Advate 9 . CHO cells produce partially processed rVWF. Therefore to obtain fully processed, mature rVWF, rVWF is exposed to recombinant CHO-cell-derived furin to remove the VWF pro-peptide 10 . Fig. 1a depicts the characteristic molecular forms of CHO-cell expressed rVWF that contain multimer species consisting of combinations of the homo- and hetero-polymers of mature and pro-VWF. For example, dimeric forms alternatively consist of two mature monomers, one mature and one pro-VWF monomer or two pro-VWF monomers, leading to the appearance of a triplet structure. This structure had been seen with previous rVWF preparations that were obtained from CHO cells 11 . As previously described, exposure to rFurin in vitro converts the heterogeneous multimer mixture of incompletely processed pro-VWF into one of highly homogeneous, structurally intact multimers 12 . This maturation process is functionally important to FVIII binding as the FVIII-binding site is located in the D'D3 domain at the N-terminus of the mature subunit 13 . In a surface plasmon resonance spectroscopic analysis using the Biacore instrument, FVIII binding to rVWF was investigated and purified VWF, as expressed by CHO cells, was compared with furin-matured rVWF. The FVIII-binding characteristics of furin-matured rVWF were very similar to those of plasma-derived VWF while pro-rVWF preparations showed a reduced FVIII-binding capacity Fig. 1b .

Fermentation for both rVWF and furin and downstream processing are performed under serum-free and protein-free conditions. After rVWF is captured by an ion-exchange column it is fully processed to mature VWF by recombinant furin. After DNA removal, rVWF is solvent-detergent (SD) treated and purified by two further chromatography steps. The rVWF resulting from the downstream process has a purity of > 99% and is thus almost pure VWF.

**Fig. 2:**
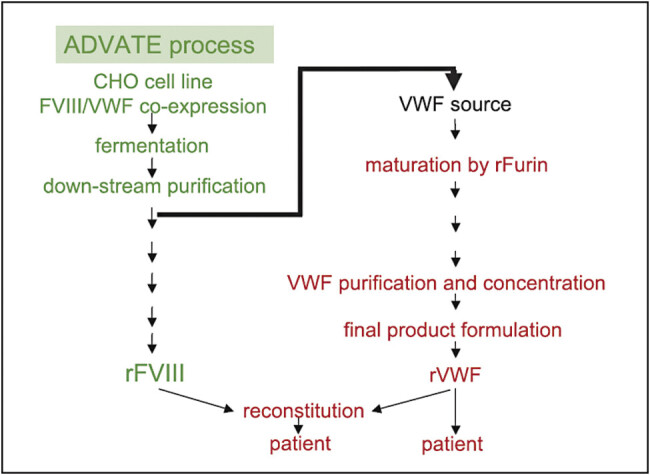
Production process of rVWF and rFVIII

Recombinant VWF is formulated in a protein-free buffer. The goal of the product development was to provide rVWF in an optimized ratio of VWF : RCo to FVIII : C to treat patients with severe von Willebrand disease (type 3). Therefore, the two lyophilized drug products, rVWF and rFVIII (Advate), are provided in individual vials, reconstituted separately and finally combined to provide a complex of rVWF and rFVIII. The schematic manufacturing process of rVWF is shown in Fig. 2

### Structure and function

The purified rVWF final drug product was extensively characterized in a number of preclinical studies with an array of state of the art biochemical and functional tests for characterizing large glycoproteins. The tests are listed in Tab. 1 . Some of the major findings from these studies are described.

**Fig. 3:**
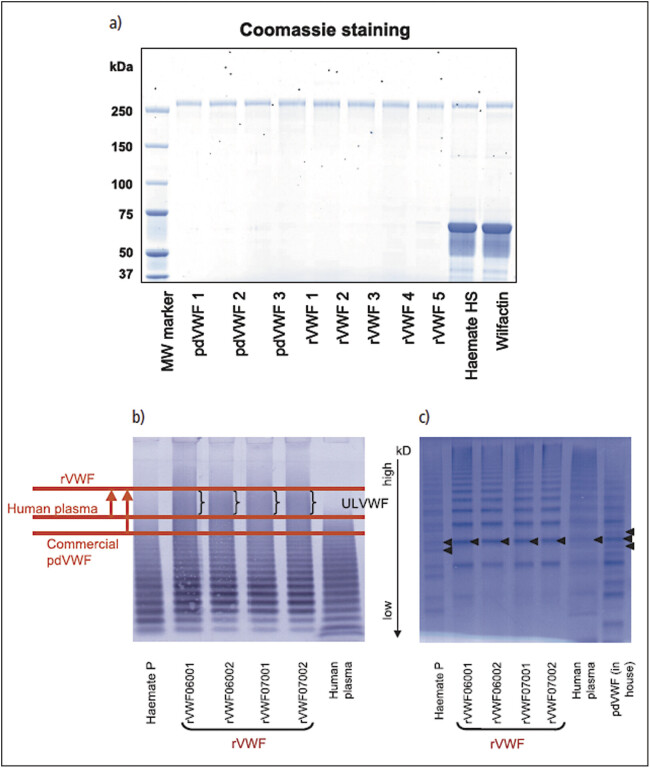
Electrophoresis of rVWF, proteins stained by Coomassie blue and agarose gel: **a)** SDS-PAGE under reducing conditions reveals the molecular integrity and protein composition of rVWF (experimental batches) compared with plasma-derived VWF (highly purified in-house preparation and two commercially available VWF-FVIII complex concentrates): Recombinant VWF shows single bands. Their migration is identical to that of the VWF bands in highly purified plasma-derived VWF. Commercial plasma-derived VWF products contain other proteins including albumin for stabilization. **b)** Agarose gel (1%): rVWF contains high and ultra-large molecular weight multimers not present in human plasma and pdVWF-FVIII concentrates (Haemate HS). rVWF batches from large scale manufacturing are shown. **c)** Agarose gel (2.5%): The absence of ADAMTS13-mediated proteolytic fragments is shown by high resolution agarose electrophoresis.

The chromatographic purification in the absence of any adjuvant protein and the final formulation results in rVWF which is a single band protein with a purity that comes close to homogeneity. The level of process-related protein impurities and host cell protein is well below the limits that are usually expected for recombinant proteins derived from CHO cells Fig. 3a a. A major characteristic of furin-matured rVWF is the homogeneity of the multimer distribution. VWF expressed by CHO cells has never been exposed to ADAMTS13. Multimer analysis showed the presence of ultra-high molecular weight multimers Fig. 3b . High-resolution multimer analysis did not reveal typical satellite bands in rVWF Fig. 3c which is evidence of rVWF’s non-exposure to limited proteolysis by ADAMTS13 similar to endothelial or platelet-derived human VWF. When rVWF is incubated with ADAMTS13, satellite bands are formed simultaneously with the disappearance of high molecular weight multimers, resulting in a multimeric pattern similar to pdVWF 14 . The FVIII-binding capacity and affinity of rVWF is comparable to that of VWF in plasma.

**Table:** **Tab. 1** Methods and tests for biochemical, structural and functional characterization of the rVWF drug product


electron microscopy protein determination molecular integrity and protein composition by electrophoretic methods multimeric structure: SDS-agarose gel systems low and high resolution peptide mapping (primary sequence) RP-HPLC analysis (fragmentation) N-terminal sequence analysis carbohydrate pattern N- and O-glycans monosaccharides sialic acids potency ristocetin cofactor activity collagen binding activity VWF antigen susceptibility to ADAMTS13 cleavage FVIII binding capacity and affinity: static and under flow binding to platelets under shear forces

**Fig. 4:**
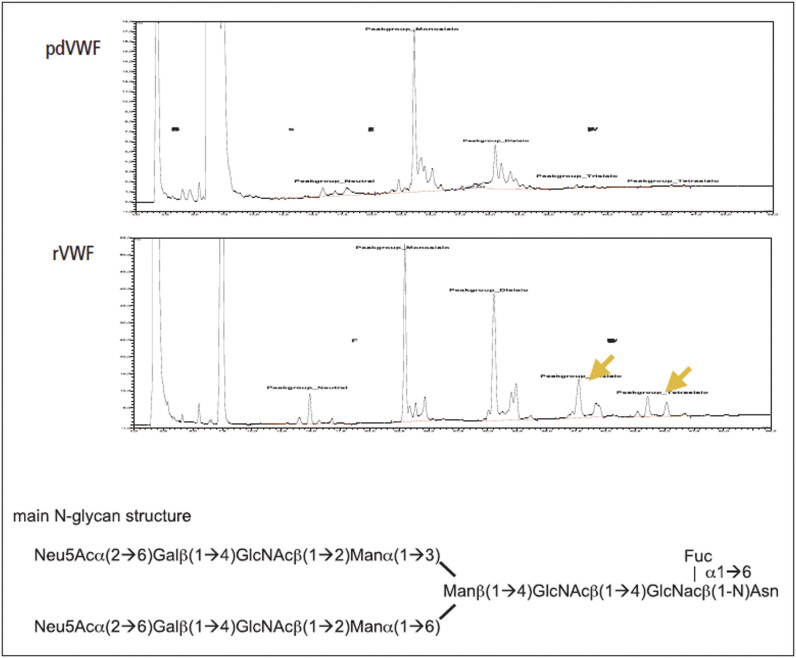
Comparison of N-glycan pattern: rVWF (lower panel) and highly purified pdVWF (upper panel) were analyzed for their N-terminal glycans by anionexchange chromatography. rVWF shows a higher proportion of sialilated tri-and tetra-antennary oligosaccharides.

Carbohydrate analysis showed an intact glycosylation pattern typical for a fully glycosylated human protein expressed in CHO cells. Similar glycosylation patterns were found for rVWF and pdVWF, which was investigated as a control. Recombinant VWF has a slightly higher proportion of sialilated tri- and tetra-antennary oligosaccharides than pdVWF, indicative of intact N-glycosides. The main glycan structure found was a biantennary di-sialilated N-glycan as also identified in pdVWF ( Fig. 4 ). Accordingly, a significantly higher content of sialic acid was found on rVWF than on plasma VWF by monosaccharide analysis. The main structure of O-glycans on rVWF is the sialilated T-antigen as present in plasma (data not shown). There was no evidence of ABO antigen structures on rVWF, which indicates that the glycosylation pattern of rVWF is similar to that of platelet-derived rVWF (data not shown).

**Fig. 5:**
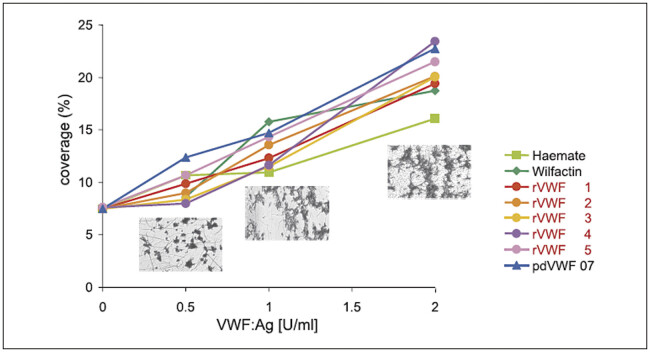
Concentration dependency of platelet adhesion to collagen fibrils under shear stress (860 s ^-1^ ) in the presence of increasing concentrations of VWF antigen

The specific activity of rVWF is higher than that of commercial plasma-derived VWF-FVIII complex concentrates, reflecting the high purity of the product and the absence of stabilizing proteins co-purified or added with plasma-derived VWF-containing products. The plasma-derived VWF that was specifically prepared as a control article for rVWF had a purity comparable to rVWF and also appeared as a single band in reducing SDS-PAGE Fig. 3a . When comparing VWF : RCo with VWF : Ag, the specific activity was higher in rVWF than in pdVWF. There was no difference between the highly purified plasma-derived VWF control article and a commercial plasma-derived VWF-FVIII complex concentrate. This reflects the high molecular weight multimers in rVWF, which bring its specific activity close to the theoretical value of one RCo U per VWF : Ag 15 , 16 . The specific activity that was found for pdVWF was in line with published results for a commercial pdVWF-FVIII complex concentrate, although a somewhat higher ratio of VWF : RCo to VWF : Ag was found previously 17 , 18 . Trace amounts of FVIII remain in the rVWF product due to the fact that it was purified from a cell culture supernatant that contains both FVIII and VWF. The higher FVIII content of the plasma-derived VWF-FVIII concentrate was found in the expected range. FVIII-binding capacity could only be measured in rVWF and the highly purified pdVWF control preparation, which both contain almost no FVIII ( Tab. 2 ). Recombinant VWF can bind to collagen under static conditions and also promote platelet adhesion to immobilized collagen under shear stress. The results are shown in Figure 5 . Five laboratory batches of rVWF were compared with three pdVWF products, two commercial VWF-FVIII complex concentrates and one highly purified VWF, used as a control preparation for the preclinical study program. No relevant differences were seen in VWF-mediated platelet adhesion between rVWF and pdVWF products.

#### In vivo characterization

**Fig. 6:**
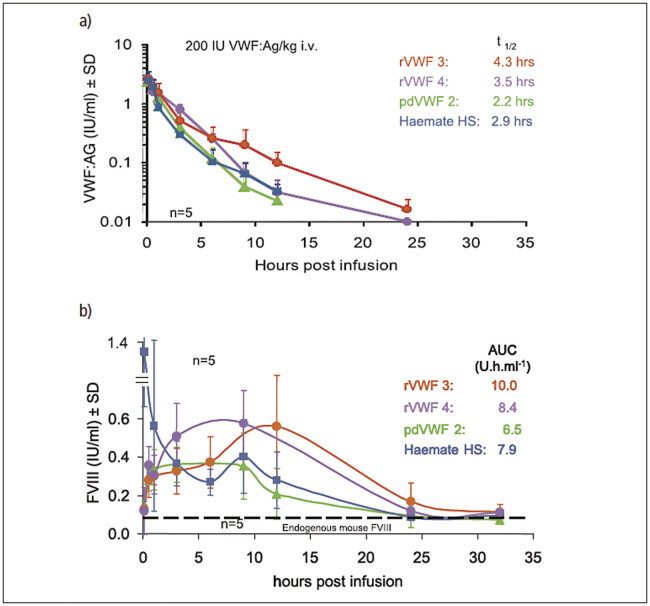
Pharmacokinetics in VWF-deficient mice **a)** rVWF and pdVWF **b)** factor VIII levels over time upon i. v. application of rVWF and pdVWF

A comprehensive study program to investigate safety and efficacy of the rVWF study drug was performed with a large variety of test models. This program took advantage of standard animal models, but also used von Willebrand-deficient pigs, dogs and knockout mice and ADAMTS13 k.o. mice. The results from the pharmacokinetic study in VWF-deficient mice are shown in Figure 6 . When two batches of rVWF were infused into VWF knock-out mice, they showed a slightly longer half-life than two pdVWF products (Haemate HS and the highly purified pdVWF control preparation). When measuring FVIII levels in the VWF knock-out mice, rVWF showed the characteristic secondary rise in endogenous FVIII levels in the VWF-deficient animals demonstrating that human rVWF is able to stabilize endogenous murine FVIII. A rise was also seen with the highly purified pdVWF control preparation but was difficult to interpret when the plasma-derived VWF-FVIII concentrate was infused due to the high amounts of FVIII that were given simultaneously with VWF contained in the complex. The slightly higher FVIII levels that were seen up to 24 hours after application in the animals treated with rVWF while animals treated with pdVWF had returned to their starting levels of FVIII also indicated the somewhat longer survival of rVWF in these animals.

## Discussion, conclusion

Replacement therapy in patients with von Willebrand disease is currently limited to plasma FVIII concentrates that also contain VWF, or purified VWF derived from human plasma 19 , 20 . So far no recombinant protein therapeutic containing VWF has been made available for treatment. We used the well-established CHO cell line, which co-expresses rFVIII and rVWF as a chaperon that facilitates and stabilizes secretion of FVIII and the cell culture supernatant obtained from it as a source for the rVWF product. An intermediate fraction from the rFVIII-PFM (Advate) 21 , 22 manufacturing process was used as a starting material to manufacture a rVWF drug product.

CHO cells do not sufficiently process pro-VWF into mature VWF and pro-peptide. Previous attempts to obtain recombinant mature VWF used CHO cell clones that co-expressed recombinant furin 23 . The process intermediate from the rFVIII-PFM manufacturing process contains a mixture of pro- and mature VWF, therefore, we developed an in vitro processing procedure with recombinant furin as the first downstream manufacturing step to obtain mature rVWF 12 , 24 . Following subsequent purification steps this drug product can be used in conjunction with the rFVIII in Advate as a complex to treat patients with severe von Willebrand’s disease (type 3) which is currently being tested in clinical trials.

Recombinant VWF is a homogenous recombinant protein with an intact multimer pattern which it retains because it is never exposed to proteases that are able to degrade it. In particular, rVWF is never exposed to ADAMTS13, which is the enzyme that by limited proteolysis generates the characteristic degradation products seen as satellite bands in agarose electrophoresis 25 . This makes rVWF similar to endothelial or platelet-derived human VWF. However, the satellite bands are formed simultaneously with the disappearance of high molecular weight multimers upon incubation with ADAMTS13 in the presence of urea under denaturating conditions. The carbohydrate pattern of rVWF is similar to that of pdVWF with respect to terminal sialic acid, indicating an intact glycosylation. Recombinant proteins derived from CHO cells with their typical glycosylation pattern have been in therapeutic use for the last two decades without any indication that the glycosylation pattern impacts safety and efficacy of such recombinant proteins.

**Table:** **Tab. 2** Product characterization of rVWF compared with commercial plasma-derived VWF-FVIII concentrate (Haemate HS) and a highly purified pdVWF produced as a reference preparation for rVWF; for comparison previoulsy published results on Haemate: *Lethagen et al. 17 , n presumably = 1; **Budde et al. 18 , n = 3; values in means ± SD where applicable; n.a.: not applicable

**Product**	**VWF:Ag protein (IU/mg) **	**VWF:RCo protein (IU/mg) **	**VWF:RCo VWF:Ag (IU/IU) **	**FVIII:c VWF:RCo (IU/IU) **	**VWF:FVIII binding capacity (%)**
rVWF (n = 5)	114 ± 5	123 ± 24	1.09 ± 0.26	0.006 ± 0.003	108 ± 19
pdVWF (n = 1)	118	59	0.50	0.070	109
Haemate	17.9 ± 4.7 n = 12	8.0 ± 1.7 n = 7	0.51 ± 0.10 n = 12	0.67 ± 0.13 n = 13	n.a.
Haemate published data	16.5 *	15.0 *	0.91 * 0.84 **	0.49 * 0.35 **	----

The specific activity, the calculated ratio between rVWF antigen content and RCo activity, is close to 1 or even above, whereas the plasma-derived sample has a much lower value. This is in accordance with the high multimer content of the rVWF preparation compared with normal plasma VWF. The functional activity of rVWF shown by the binding capacity for FVIII and VWF : RCo activity was found to be similar to or higher than that of the in-house purified or commercial pdVWF products. The primary hemostatic activity of rVWF was demonstrated by VWF-induced platelet adhesion under high shear stress. Pharmacokinetics of rVWF and pdVWF are different in VWF-deficient mice: Recombinant VWF has a slightly longer half-life than the pdVWF Haemate HS and a highly purified pdVWF control. This is consistent with previous observations for another rVWF product of substantially longer half-lives in dogs and pigs with severe von Willebrand deficiencies. However, the rVWF product used in the previous studies also contained heteropolymers of pro- and mature VWF and therefore is not directly comparable to the current product 26 , 27 , 28 , 29 , 30 , 31 . It remains to be demonstrated if similar differences will be seen in primate studies and in the clinic. Preparations of rVWF stabilized endogenous murine FVIII in the circulation of VWF-deficient mice. This secondary rise of endogenous FVIII levels indicates that a clinically and therapeutically important feature of VWF has been retained in the recombinant molecule.

## Notes

The similarity of rVWF’s in vitro and in vivo properties to those of pdVWF facilitated the decision to develop the first recombinant entity that has the potential to treat patients with VWD.
